# Pathogenic genes implicated in sleep-related hypermotor epilepsy: a research progress update

**DOI:** 10.3389/fneur.2024.1416648

**Published:** 2024-06-20

**Authors:** Yufang Yang, Jinmei Tuo, Jun Zhang, Zucai Xu, Zhong Luo

**Affiliations:** ^1^Department of Neurology, Affiliated Hospital of Zunyi Medical University, Zunyi, China; ^2^Department of Nursing, Affiliated Hospital of Zunyi Medical University, Zunyi, China

**Keywords:** sleep-related hypermotor epilepsy, genes, mutations, pathogenesis, research progress

## Abstract

Sleep-related hypermotor epilepsy (SHE) is a focal epilepsy syndrome characterized by a variable age of onset and heterogeneous etiology. Current literature suggests a prevalence rate of approximately 1.8 per 100,000 persons. The discovery of additional pathogenic genes associated with SHE in recent years has significantly expanded the knowledge and understanding of its pathophysiological mechanisms. Identified SHE pathogenic genes include those related to neuronal ligand- and ion-gated channels (*CHRNA4, CHRNB2, CHRNA2, GABRG2*, and *KCNT1*), genes upstream of the mammalian target of rapamycin complex 1 signal transduction pathway (*DEPDC5*, *NPRL2*, *NPRL3*, *TSC1*, and *TSC2*), and other genes (*CRH*, *CaBP4*, *STX1B*, and *PRIMA1*). These genes encode proteins associated with ion channels, neurotransmitter receptors, cell signal transduction, and synaptic transmission. Mutations in these genes can result in the dysregulation of encoded cellular functional proteins and downstream neuronal dysfunction, ultimately leading to epileptic seizures. However, the associations between most genes and the SHE phenotype remain unclear. This article presents a literature review on the research progress of SHE-related pathogenic genes to contribute evidence to genotype–phenotype correlations in SHE and establish the necessary theoretical basis for future SHE treatments.

## 1 Introduction

**Figure 1:**
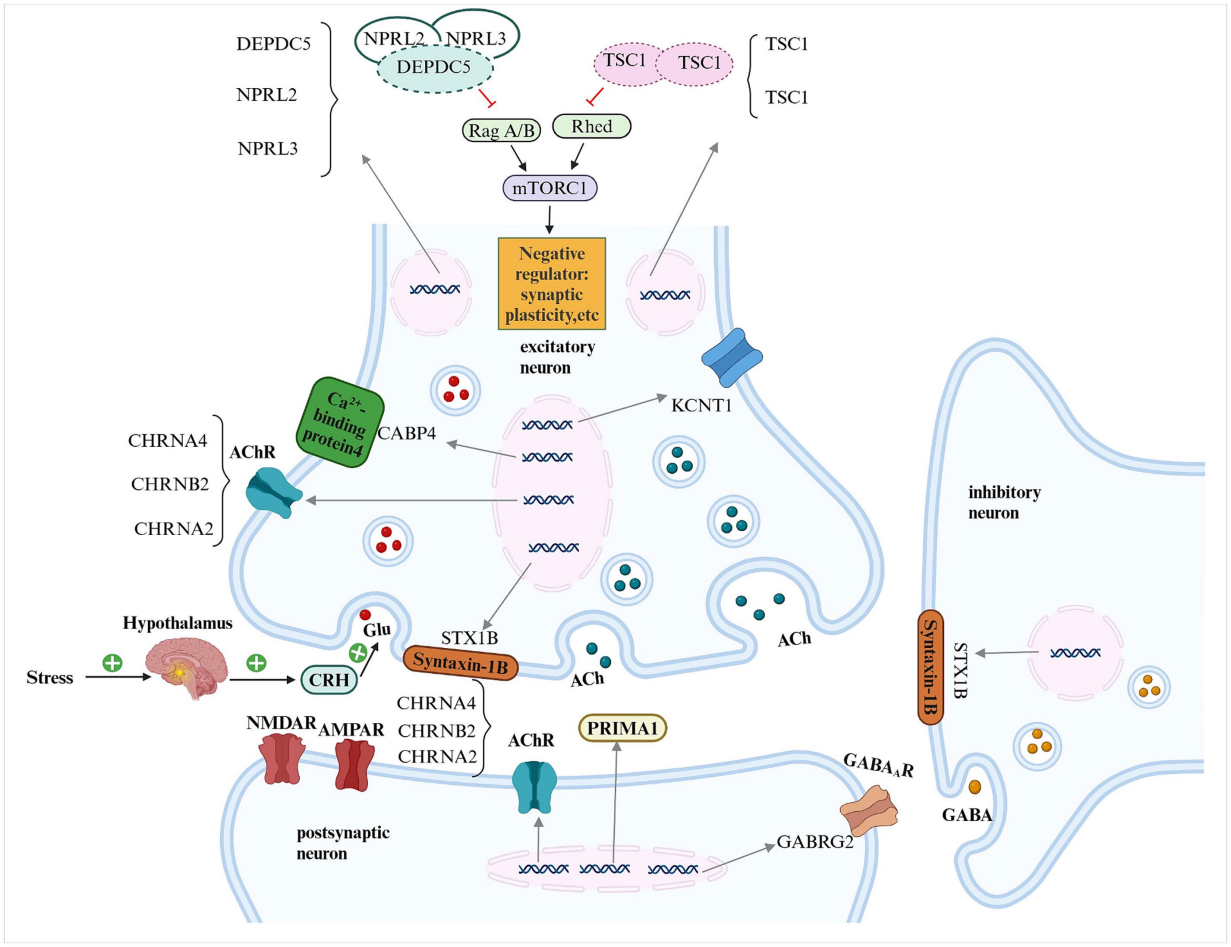
Schematic representation of the pathogenic genes implicated in sleep-related hypermotor epilepsy (SHE) and their potential mechanisms DEPDC5, DEP domain-containing protein 5; NPRL2, NPRL3, nitrogen permease regulator-like 2, 3; TSC1, TSC2, tuberous sclerosis complex 1, 2; mTORC1, mammalian target of rapamycin complex 1; ACh, acetylcholine; AChR, acetylcholine receptor; CHRNA4, CHRNB2, CHRNA2, cholinergic receptor nicotinic alpha 4, beta 2 and alpha 2 subunits; KCNT1, potassium sodium-activated channel subfamily T member 1; CRH, corticotropin-releasing hormone; Glu, glutamate; NMDAR, n-methyl-d-aspartate receptor; AMPAR, alpha-amino-3-hydroxy-5-methyl-4-isoxazole propionic acid receptor; GABRG2, gamma-aminobutyric acid type A receptor, subunit gamma 2; PRIMA1, Proline-Rich Membrane Anchor 1; GABA_A_R, γ-aminobutyric acid type A receptor; GABA, γ-aminobutyric acid.

Epileptic seizures are classified as focal, generalized, or unclassified, based on their origin, or as motor or non-motor seizures, based on their motor manifestations. Similarly, epileptic syndromes are classified into specific and nonspecific types according to their clinical features or into genetic, structural, metabolic, immune-related, or unclassified epileptic syndromes, based on their etiology. According to the 2022 International League Against Epilepsy (ILAE) Classification of Epilepsy Syndromes, sleep-related hypermotor epilepsy (SHE) is identified as a focal epilepsy syndrome with genetic, structural, or genetic structural etiology, characterized by hypermotor seizures that typically present as brief (<2 min), clustered “hypermotor stereotyped episodes” with sudden onset and offset during non-rapid eye movement (NREM) sleep, particularly stage II (1, 2). Despite extensive research, the pathogenesis of SHE remains unclear owing to its complexity. Genetic mutations constitute the most popular etiology of SHE in the current literature, with identified SHE-related pathogenic genes including neuronal ligand-gated and ion-gated channel-related genes (*CHRNA4*, *CHRNB2*, *CHRNA2*, *KCNT1*, *GABRG2*), upstream genes in the mammalian target of rapamycin complex 1 (mTORC1) signaling pathway (*DEPDC5*, *NPRL2*, *NPRL3*, *TSC1*, *TSC2*), and other genes (*CRH*, *CaBP4*, *STX1B*, *PRIMA1*). Genetic mutations in the nicotinic acetylcholine receptor (nAChR) subunit are most closely associated with SHE, and patients carrying these genetic mutations often exhibit classical SHE symptoms. Notably, genes associated with the GATOR1 complex, particularly *DEPDC5*, exhibit the highest mutation frequency. These gene mutations often result in hereditary structural abnormalities such as focal cortical dysplasia, what’s more, high drug resistance, diurnal seizures, and incomplete penetrance. *KCNT1* mutations demonstrate a broad phenotypic spectrum among carriers and are often associated with severe epilepsy phenotypes, such as intellectual disability and mental and behavioral abnormalities. *CRH* mutations appear to increase susceptibility to epilepsy and may serve as a trigger for epileptic seizures. Only one case of each of the *CaBP4, STX1B*, and PRIMA1 mutations has been reported globally to date; therefore, additional clinical validation is required to determine their pathogenic roles in SHE. Multiple studies have investigated SHE-related genes; nevertheless, the specific mechanisms by which these genetic mutations contribute to the occurrence and development of SHE remain unclear, and clinical treatment of SHE remains limited. This study reviewed the genetic mutations associated with SHE and their possible pathogenic mechanisms (Table 1; Figure 1), aiming to provide insight for future research on the pathogenesis of SHE and its diagnostic and therapeutic measures.

**Table 1:** Confirmed and potential pathogenic genes and mutations in sleep-related hypermotor epilepsy (SHE).

Gene (references)	Mutation	Mutation type	Location	Heritance	Function effects	Pathomechanism	Clinical features
CHRNA4 (3–6)	I275F S280F S284W T293I S252L S252F	Missense	M2	ADSHE	1. Increased sensitivity to ACh 2. Increased receptor desensitization to ACh 3. Reduced Ca2+ permeability 4. Reduced Ca2+ dependence of the ACh response	Gain-of-function	Typical SHE
	R336H		Second intracellular domain	NA		NA	
	G307V R308H R483W S252L I248F		NA	NA		NA	
	G507R R360Q		NA	NA		NA	
	L291dup	Insertional	M2	NA		NA	
	D190D I265I	Synonymous	NA	NA		NA	
	S284L/S248F	Missense	M2	ADSHE		Gain/loss-of-function	
CHRNB2 (3)	V287L V287M	Missense	M2	ADSHE	1. Retardation of channel desensitization 2. Increased ACh sensitivity	Gain-of-function	Psychological morbidities
			
	I312M	Missense	M3	ADSHE	Increased ACh sensitivity	Gain-of-function	Typical SHE with distinct memory deficits
	L301V V308A			NA			Typical SHE
	V337G		Intracellular domain in M3-M4	ADSHE	NA	NA	Sporadic SHE
CHRNA2 (7–9)	I279N	Missense	M1	NA	Increased nAChR sensitivity to agonists	Gain-of-function	Typical SHE with ictal fear and nocturnal wandering
	I297F		M2	NA	Reduced current expression	Loss-of-function	Typical SHE
	Y252H		N-terminal ligand-binding domain	NA	Reduced numbers of channels bound to agonists		
GABRG2 (10)	T90M	Missense	Extracellular N-terminal domain	NA	1. Decreased GABA-evoke current 2. Reduced synaptic clustering and distribution of GABA_A_R	NA	Paroxysmal dystonia
	Q217X			NA			Typical SHE
	T317N		M2	NA			Moderate intellectual disability
KCNT1 (11–16)	R928C	Missense	Within or flanking the NAD + -binding domain	ADSHE	Alter channel-channel interactions	Gain-of-function	1. Early onset age, severe phenotype, 2. Intellectual disability and psychiatric feature 3. The same mutation causes different epilepsy types
	Y796H			ADSHE			
	M896I			Sporadic SHE			
	R398Q		S6-RCK1 in C-terminal domain	ADSHE			
	G288S		S5	ADSHE			
	A934T		RCK2	SHE			
	R950Q			ADSHE	NA		With FCD type Ib, incomplete penetrance
	R933C			ADSHE			The same mutation causes different epilepsy types
DEPDC5 (17–19)	Arg422* 193 + 1G > A Arg389Profs*2 etc. Have no hot-spot mutating	Nonsense is the most common	NA	Sporadic familial SHE	mTORC1 hyperactivates mTOR pathway deregulates	Haploid dosage insufficient OR loss-of-function	Mostly focal seizure, high drug resistance. Often with a brain malformation
NPRL2 (17, 20–22)	Leu105Pro	Missense	NA	ADSHE		Loss-of-function	NA
	Arg34*	Nonsense	NA	ADSHE		NA	
	CNV Exon2-11	Copy number variation	NA	Sporadic SHE			
NPRL3 (17, 20, 23–25)	Ser279Phefs*52 Glu508Argfs*46	Frameshift	NA	NA			
	GIn188* Arg424* Tyr519*	Nonsense					
TSC1 (26)	Ser282GInfs*36	Frameshift	NA	NA	mTOR cascade dysfunction	NA	NA
TSC2 (19)	Arg905GIn	Missense	NA	NA	Haploid dosage insufficient caused mTORC1 hyperactivate	Loss-of-function	Refractory SHE possibly generalized focal seizure
CRH (27, 28)	g.1470C > A	Nucleotide variation in promoter	NA	Familial ADSHE	Gene expression levels change	NA	Typical SHE
	g.1166G > C		NA	Sporadic ADSHE			
	Pro30Arg	Missense	Coding region of the CRH	NA	Protein secretion levels decrease in a short time		
CaBP4 (29)	G155D	Missense	N-lobe of CaBP4 within EF1	Familial ADSHE	Decreases ion channel activation Reduced Ca2 + concentration	NA	Typical SHE
STX1B (30)	c.106-2A > G	Splice-site	NA	NA	NA	NA	Typical SHE with peri-ictal hypotension
PRIMA1 (31)	c.93 + 2 T > C	Splice-site	NA	ARSHE	Knock out of PRIMA1 Reduce AChE and increase ACh accumulation in the synapse	NA	Often with incontinence, postictal confusion, and moderate intellectual disability

CHRNA4, CHRNB2, CHRNA2, cholinergic receptor nicotinic alpha 4, beta 2 and alpha 2 subunit; GABRG2, gamma-aminobutyric acid type A receptor, subunit gamma 2; KCNT1, potassium sodium-activated channel subfamily T member 1; DEPDC5, DEP domain-containing protein 5; NPRL2, NPRL3, nitrogen permease regulator-like 2, 3; TSC1, TSC2, tuberous sclerosis complex 1, 2; CRH, corticotropin-releasing hormone; CaBP4, Calcium-binding protein 4; STX1B, Syntaxin 1B; PRIMA1, Proline-Rich Membrane Anchor 1; M1, 2, 3, transmembrane domains 1, 2, 3; RCK2, regulatory domains conducting potassium ions 2; S5, S6, transmembrane domain 5, 6; ADSHE, ARSHE, autosomal dominant, recessive sleep-related hypermotor epilepsy; ACh, acetylcholine; AChR, acetylcholine receptor; GABA_A_R, γ-aminobutyric acid type A receptor; mTOR, mTORC1, mammalian target of rapamycin, complex 1; AChE, acetylcholinesterase; FCD, focal cortical dysplasia.

## 2 Ion channel-related pathogenic genes

### 2.1 Nicotinic acetylcholine receptor-related genes

Neuronal nicotinic acetylcholine receptors (nAChRs) comprise a variety of subunits, forming different subtypes, which are widely distributed in the presynaptic, postsynaptic, and non-synaptic regions of the mammalian brain (32). nAChRs are crucial for the regulation of neural functions including neuronal activity, neurotransmitter release, and synaptic plasticity (33).

Different nAChR subtypes are distributed in different brain regions and neuron types (33). The most abundant nAChR subtypes in the thalamus and cerebral cortex are (α4)_2_, (β2)_3_, and (α7)_5_. Ligand-binding sites across different receptor types are predominantly located at the extracellular domains of α subunits [primarily α4 in the central nervous system (CNS)] and complementary binding surfaces of β subunits (primarily β2 in the CNS). Upon ligand binding, nAChRs modulate neuronal excitability and the release of various neurotransmitters in different brain regions by regulating the influx of cations across the cell membrane, influencing various physiological and behavioral activities, such as synaptic plasticity, motor function, attention, learning, and memory (34, 35). Among the genes implicated in SHE pathogenicity, those that encode nAChR subunits are among the most popular in the literature. Approximately 12% of autosomal dominant SHE (ADSHE) families carry pathogenic variants of genes encoding three nAChR subunits (*CHRNA4, CHRNB2*, and *CHRNA2*) (3, 36, 37). Among them, mutations in CHRNA4 and CHRNB2 are associated with autosomal dominant and sporadic SHE, whereas mutations in CHRNA2 are only associated with the autosomal dominant subtype.

Overall, 17 *CHRNA4* mutations have been identified in patients with SHE to date, comprising missense mutations (S248F, I275F, S280F, S284L, S284W, T293I, R336H, G307V I265I, R308H, R483W, S252L, I248F, G570R, R360Q), an insertion mutation (L291dup), and a synonymous mutation (D190D) (3–6). Most mutations alter the transmembrane domain 2 (TM2), which constitutes the main pore-forming region of nAChR subunits, leading to a functional gain of the nAChR (3–6). The six confirmed CHRNB2 mutations (V337G, V287L, V287M, I312M, L301V, V308A) are all missense mutations (3), mainly altering the TM2-TM3 or TM3 regions. The TM2-TM3 region plays a crucial role in controlling the opening and closing of the nAChR. The three CHRNA2 mutations (I279N, I297F, and Y252H) are all missense mutations (7–9), which alter the TM1, TM2, and N-terminal ligand-binding domain, respectively.

Studies have shown that mutations in genes encoding nAChR subunits typically result in enhanced receptor function. Studies using transgenic rats (V286L-TG, corresponding to the human β2-V287L mutation), Xenopus oocytes, and mammalian cells have found that mutations increase the function of the α4β2*nAChR, with the gain of function manifesting as increased sensitivity of the receptor to agonists and delayed desensitization (3, 38, 39). Multiple studies have attempted to explain the increased receptor function. Mutations may change the ratio of nAChR subunits or reduce the allosteric enhancement caused by Ca ± concentration, resulting in enhanced glutamate release during the synchronous discharge of pyramidal neurons (38). In addition, hyperfunctional α4β2-nAChR may enhance the stimulation of micro-awakening events caused by transient β2-nAChR activity due to low-level ACh release during NREM sleep, thereby leading to typical hyperexcitement and sudden awakening during NREM sleep (38). Gain-of-function receptors change the excitability of thalamo-neocortical circuits through multiple mechanisms, resulting in epileptic seizures, as follows: (1) rebound excitation of pyramidal cells: thalamocortical neurons are inhibited during slow-wave sleep following excessive acetylcholine release combined with the mutated hyperfunctional nAChR, resulting in excessive γ-aminobutyric acid (GABA) release in the prefrontal cortex. This results in abnormal hyperpolarization of pyramidal neurons, which leads to low-threshold voltage-gated calcium channel closure and H-current increase, increasing pyramidal cell susceptibility to rebound excitation after inhibition (40). (2) thalamocortical interactions: sleep spindles generated in the thalamus during stage II NREM sleep are transmitted to thalamocortical neurons through the modulation of thalamic reticular nucleus cells (41), resulting in cortical neuron synchronization in the thalamocortical region. Sleep spindle oscillations can transform into epileptiform activity, attributed to glutamate release into thalamic reticular cells through nAChR-dependent stimulation and subsequent inhibition of GABA release from the thalamic reticular cells (42). (3) GABAergic interneurons: animal studies have shown that GABAergic interneurons innervate pyramidal cells carrying α4β2-nAChR in layers II/III of the cerebral cortex and that genetic mutations can alter receptor function, thereby activating a larger number of interneurons. The role of GABAergic interneurons in the cerebral cortex remains unclear; nevertheless, this mechanism may facilitate pyramidal cell firing synchronization after recovery from inhibition caused by interneurons, the spreading of local electrical activity to other areas, and ultimately epileptic seizures (40).

Moreover, mutations in nAChR subunit-related genes can lead to loss of receptor function, resulting in diseases. Expressing the human *CHRNA4* S284L mutant in transgenic rats (S286L-TG) demonstrated that loss of function in the S286L mutant α4β2-nAChR manifests as impaired GABAergic inhibition in the motor thalamic nuclei and enhanced glutamatergic transmission through the thalamocortical motor pathway (from the motor thalamic nucleus to the secondary motor cortex), leading to paroxysmal nocturnal wandering and nocturnal arousal (43, 44). Furthermore, enhanced glutamatergic transmission through the thalamic hypermotor pathway (from the motor thalamic nucleus to the subthalamic nucleus) leads to nocturnal paroxysmal dystonia (43, 44). On the other hand, this loss of function manifests as upregulation of the MAPK/Erk signaling pathway in the secondary motor cortex and thalamus. The upregulated signaling pathway combined with high glutamatergic transmission (repeated/sustained propagation of discharges) results in the upregulation of astrocyte Cx43 (45). In cases of abnormal receptor function, the propagation of physiological (sleep spindle bursts) and pathological (interictal/ictal discharges) discharges to the orbitofrontal cortex (OFC) and the excitatory tripartite synaptic transmission induced by Cx43 upregulation are enhanced, resulting in epileptic seizures during NREM sleep and subsequent frequent epileptic seizures during the same night (45). In addition, the weakened transmission of the thalamocortical cognitive pathway in S286L-TG may lead to cognitive impairment in patients with SHE (36). Mutant α4β2-nAChR is more susceptible to acetylcholine activation induced by pathological thalamocortical oscillations during stage II NREM sleep, which may explain the typical occurrence of SHE during this period (46).

Individuals carrying CHRNA4, CHRNB2, or CHRNA2 mutations typically exhibit classic SHE seizures. Additionally, patients carrying CHRNA4 or CHRNB2 mutations have a higher frequency of cognitive impairment, intellectual disability, and schizophrenia-like symptoms (47), whereas patients carrying CHRNA2 mutations exhibit paroxysmal arousal, occasional nocturnal wandering, and episodic panic attacks; however, these seizures do not correlate with specific sleep stages, which may be related to α2-nAChR distribution in the brain (2). What’s more, Patients with CHRNA2 mutations demonstrate increased sensitivity to treatment with clonazepam or oxazepam (8).

Carbamazepine selectively inhibits nAChR and is the first choice anti-epileptic drug for patients with SHE (48); however, approximately one-third of patients develop drug resistance (48), speculating that the drug may only be effective for receptors with gained function. In S286L-TG with loss of α4β2-nAChR function, a certain concentration of zonisamide inhibits Cx43 activity and expression in the plasma membrane of astrocytes and enhances receptor agonist-induced GABA release (36, 45), which relieves epileptic seizures in SHE model rats. However, no clinical studies have been conducted on humans. Studies have found that fenofibrate, a peroxisome proliferator-activated receptor α (PPARα) agonist, can be used as a negative regulator of nAChR and is effective for patients with CHRNA4 and CHRNA2 mutant and non-mutant SHE (49).

### 2.2 GABA receptor-related genes

The GABA type A receptor (GABAAR) is a chloride channel that serves as the principal inhibitory receptor in the mammalian CNS. Upon binding to the inhibitory neurotransmitter GABA, it becomes activated, facilitating Cl^−^ influx and the generation of hyperpolarizing potentials. Subsequently, it mediates rapid GABAergic inhibition and modulates excitability in the cerebral cortical network (50, 51). The disruption or weakening of GABAAR-mediated inhibition, which increases neuronal excitability, is an important pathophysiological basis for the development of various epilepsy syndromes. The γ2 subunit is a crucial component of the GABA_A_R and is essential for cell membrane receptor trafficking, synaptic site receptor clustering and maintenance, and current dynamic modulation (52). *GABRG2*, the gene encoding the γ2 subunit, is widely expressed in the CNS and is most closely related to genetic epilepsy among the genes encoding GABAAR subunits. *GABRG2* mutations primarily manifest as febrile seizures and developmental and epileptic encephalopathy (50).

A Chinese series study of 58 unrelated patients (10) detected three rare *GABRG2* variants (T90M, T317N, and Q217X) in three distinct probands. T90M and Q217X were transmitted through an unaffected mother and father, respectively, whereas T317N was a *de novo* mutation. The age of onset of epilepsy in these patients was 7 and 12 years, respectively, and the seizure onset time was <1 min. All patients exhibited hyperkinetic seizures. Patients carrying T90M and T317N mutations experienced paroxysmal awakening at night, and one patient with the T317N mutation experienced diurnal seizures, with loss of consciousness and resistance to drug treatment. Functional studies and analyses indicate that T90M and T317N impair GABA-mediated synaptic inhibition by disrupting receptor surface expression, causing endoplasmic reticulum retention and channel gating defects through various mechanisms, thereby lowering the threshold for epileptic seizures. Q217X disrupts synaptic site receptor clustering and distribution; however, its pathogenic significance remains unknown (10). Further studies are required to determine whether this mutation alters GABA_A_R recruitment and subunit assembly and clarify whether the mutant subunit leads to dominant negative inhibition and cytotoxic effects, thereby causing loss of receptor function. *GABRG2* mutations may lead to SHE pathogenicity by reducing synaptic expression and receptor clustering, resulting in channel dysfunction and GABAergic transmission impairment within cortical circuits.

Three patients with each of the T90M, T317N, and Q217X mutations demonstrated clinical manifestations of classic SHE. Cohort studies and literature reviews showed that the GABRG2 mutation genotype was not associated with a specific phenotype; however, the location of the mutation on the protein and the extent of channel functional impairment induced by the mutation might be related to disease severity (50, 53). Further studies should clarify whether the distribution of GABRG2 mutations in brain regions is associated with SHE. Levetiracetam and carbamazepine are suitable for patients with T90M and Q217X mutations, whereas patients with T317N who have diurnal epileptic seizures are resistant to carbamazepine and lamotrigine. Several studies have shown that the pathogenic mechanism of GABRG2 mutation constitutes the combined effect of reduced channel function and cell homeostasis caused by the presence of mutant proteins (54). Therefore, enhancing the function of wild-type GABA receptor channels by preventing the production and promoting the clearance of mutant proteins may improve disease severity in these patients.

### 2.3 Na+ -activated K+ channel-related genes Slo2.2

The Na+ -activated K+ channel Slo2.2 is encoded by KCNT1 and is widely expressed in neurons in the brainstem, frontal cortex, and hippocampus. This receptor plays an important role in regulating neuronal excitability by stabilizing resting membrane potential, participating in action potential repolarization, and modulating adaptive discharge patterns by generating slow afterhyperpolarization potentials and depolarizing afterpotentials (55). KCNT1 mutations can cause a range of epilepsy and neurodevelopmental disorders.

A large-scale study of *KCNT1*-related epilepsy involving 248 individuals showed that the epilepsy phenotypes caused by *KCNT1* mutations primarily included early epilepsy of infancy with migrating focal seizures (EIMFS), developmental and epileptic encephalopathy other than EIMFS, ADSHE or sporadic SHE, temporal lobe epilepsy, and other types of epilepsy with tonic–clonic seizures (56, 57). In that study, 53 patients presented with SHE. More than 60 pathogenic variants of *KCNT1* have been reported to date, all of which are missense mutations. *KCNT1* mutations associated with SHE include R928C, Y796H, R398Q, M896I, A934T, R950Q, G288S, and R933C (11–16). These mutations are all missense mutations, and the mutations are mainly concentrated in the NAD+ binding domain and the RCK2 domain (56). Patients with these *KCNT1* mutations tend to exhibit an earlier age of onset (20 months to 9 years), a higher incidence of early-onset refractory epilepsy in their family history, higher rates of comorbid intellectual disabilities and behavioral abnormalities, higher drug resistance rates, and more complete penetrance than patients with nAchR mutant-derived SHE (12, 14). A previous study on four patients carrying KCNT1 mutations found that they all had a mild malformation of cortical development (mMCD). Three patients with negative magnetic resonance imaging (MRI) results were pathologically diagnosed with type I focal cortical dysplasia (FCDIa/b) after epilepsy surgery, and one patient showed periventricular nodular ectopia on MRI (14). The patient with pathological manifestations of FCDIb had significantly improved epileptic seizures postoperatively. In contrast, the two patients with FCDIa had the same epileptic seizures as those preoperatively at different intervals postoperatively (14). The authors speculated that the epileptogenicity of KCNT1 mutations may be caused by both excitatory disorders controlling Na+-K+ transport and mMCD. The poor surgical results of the two patients with FCDIa may be related to the diffuse nature of FCDI and the broader epileptogenic network caused by germline KCNT1 mutations. The persistent epileptogenicity of the mutation postoperatively maintains the tendency to trigger epileptic seizures (14). The early age of onset and comorbid intellectual disability in patients with KCNT1 mutations may be attributed to abnormal synaptic development and synaptic plasticity (58) or altered ability of the C-terminal domain of the Slo2.2 channel to interact with the fragile X mental retardation protein (59) caused by the mutation.

Studies on Xenopus oocytes and human embryonic kidney (HEK) cell expression found that SHE-related pathogenic KCNT1 mutations enhance the Na sensitivity of the channel by inhibiting subconductance states, enhancing channel cooperativity, reducing single-channel conductance, and altering current–voltage relationships and biochemical interactions with the binding partners, thereby leading to a gain of channel function (60). A study on KCNT1 gain-of-function variant mouse models found that gain-of-function mutations reduce the excitability of cortical GABAergic neurons and increase homotypic synaptic connections, leading to excitatory/inhibitory imbalance, network hyperexcitation and epileptic seizures (61). These Slo2.2 gain-of-function mutations are primarily expressed in inhibitory CNS interneurons and result in excessive K^+^ conductance, which inhibits the inhibitory interneurons, preventing the inhibition of certain neural circuits and ultimately resulting in overall neural circuit hyperexcitability, which manifests as seizures (62). In summary, the mechanism by which *KCNT1* mutations cause SHE may involve increased firing frequency of pyramidal neurons due to enhanced IK_Na_, resulting in an accelerated repolarization rate of action potentials and weakened inactivation of Na+ channels, which promote high-frequency firing of pyramidal neurons. Alternatively, a reduction in excitatory inhibition of interneurons may occur. Studies have shown that KCNT1 is also expressed in interneurons and that the slow accumulation of sodium ions in fast-spiking interneurons can stimulate KCNT1 to inhibit their excitability. These effects are enhanced in the presence of mutant KCNT1 channels, resulting in network disinhibition and causing epileptic seizures (63). Mouse model studies utilizing the human *KCNT1* gain-of-function mutations Y796H and L456F confirmed this mechanism. Meanwhile, research on the human KCNT1 gain-of-function homozygous P924L mutation expressed in induced pluripotent stem cell-derived neurons revealed that the mutation increased the number of action potentials and firing rate, increasing the excitability of excitatory neurons (64). Despite variations in findings, all studies concluded that *KCNT1* mutations alter neuronal excitability.

Some mutations, such as G288S and R398Q, can cause both ADSHE and EIMFS, and even coexist (16, 65). This may indicate that KCNT1 mutations have pleiotropic effects (modifying genes, environmental factors, etc.). Therefore, determining the genotype-phenotype correlation of KCNT1 mutations proves difficult. Considering the gain-of-function nature of KCNT1 mutations, potassium channel blockers, such as quinidine, may serve as a precision therapeutic option for KCNT1 mutation-associated SHE; however, conflicting reports exist on quinidine’s efficacy in treating SHE (66, 67), and its significant cardiac risks introduce substantial challenges. Further research is necessary to determine the efficacy and safety of quinidine in KCNT1 mutation-associated SHE.

## 3 mTORC1 signaling pathway-related pathogenic upstream genes

### 3.1 GATOR1 complex-related genes

The mechanistic target of rapamycin complex 1 (mTORC1) signaling pathway is a major regulator of cellular metabolism. mTORC1 plays a crucial role in synaptic transmission, plasticity, neural network activity, and neurogenesis in the CNS (68). The GATOR1 complex, an upstream repressor of the mTORC1 signaling pathway, inhibits the transmission of mTORC1 signaling by acting as a GTPase-activating protein (GAP) of Rag A/B, thereby affecting brain development and function. The complex comprises three stably interacting subunits: DEP domain-containing protein 5 (DEPDC5), nitrogen permease regulator-like 2 (NPRL2), and nitrogen permease regulator-like 3 (NPRL3). Mutations in GATOR1-RagA/B-mTORC1 pathway-related genes (*DEPDC5*, *NPRL2*, and *NPRL3*) have been reported in over 180 families with focal epilepsy, with *DEPDC5* mutations being the most common, accounting for 85% of all cases (17). The predominant focal epilepsy phenotype associated with GATOR1 gene mutations was SHE, accounting for 25% (45/183) of cases. In addition, functional studies revealed that *DEPDC5* was mainly a loss-of-function variant (67%) (17, 69). Loss of function leads to excessive mTORC1 pathway activation, which disrupts the formation of neural circuits and alters established neural networks, thereby promoting epilepsy (70). In addition to epilepsy, GATOR1 pathogenic variants can cause malformations in cortical development, neurodevelopmental disorders, and neuropsychiatric conditions.

*DEPDC5* encodes the DEP domain-containing protein 5 (DEPDC5), which is the largest subunit of the GATOR1 complex. Mutations in this gene are primarily associated with focal epilepsy (mainly SHE), cortical developmental malformations (mainly FCD type II), and sudden unexpected deaths in epilepsy (SUDEP) (71). The NPRL2 and NPRL3 genes encode nitrogen permease regulator-like 2 protein (NPRL2) and nitrogen permease regulator-like 3 protein (NPRL3), respectively. *DEPDC5, NPRL2*, and *NPRL3* are expressed in human brain regions, including the frontal, temporal, parietal, and occipital lobes (17). The three proteins interact to form GATOR1, and mutations in any of the three implicated genes can lead to mTORC1 signaling pathway overactivation (20).

A cohort study of 30 unrelated ADSHE families identified four *DEPDC5* loss-of-function mutations in four families (4/30, 13%), totaling nine patients carrying *DEPDC5* mutations. Among them, seven patients exhibited drug resistance, with a drug resistance rate as high as 78%. Among these drug-resistant patients, six experienced diurnal seizures (72). Next-generation sequencing analysis of genes of 404 unrelated families with familial and sporadic focal epilepsy identified seven mutations in 17 of 91 patients with SHE. These mutations have also been reported in other focal epilepsies, such as temporal lobe epilepsy, and in disease-free individuals (20), demonstrating the phenotypic heterogeneity and incomplete penetrance of *DEPDC5* mutations. A clinical and genetic study of 73 patients with focal epilepsy (both familial and sporadic) with GATOR1-related gene mutations revealed 22 *DEPDC5* mutations in 26 patients with SHE (17). These were mainly nonsense mutations, accounting for >60%. Six patients had FCD on MRI, and 12 inherited the mutation from unaffected parents. *DEPDC5* mutation-related SHE was also associated with a high drug resistance rate (13/22, 59%), incomplete penetrance, diurnal onset (3/26), FCD (primarily FCDIIb) (6/26), and SUDEP (6/26), with the latter possibly associated with early epilepsy onset, high drug resistance rates, and predominantly nocturnal seizures in patients (17). Genetic mutation analysis of 103 cases of SHE (16 familial and 87 sporadic) revealed a *DEPDC5* mutation rate of 3.9%, which was the highest mutation rate observed in this study. Among the four identified loss-of-function mutations, three were observed in sporadic SHE cases who presented with FCD (21), suggesting that *DEPDC5* mutations contribute to FCD etiology (18) and that the occurrence of SHE may be related to hereditary brain structural lesions. A case series reported three different *DEPDC5* mutations in eight patients with SHE. These patients were relatively older (aged 46, 59, and 63 years), one patient had a history of severe depression, and two patients had refractory epilepsy (73). The DEPDC5 mutation rate was the highest in this study, consistent with previous studies (21); however, the number of included cases was limited. A study of 16 patients with *DEPDEC5* mutation-related epilepsy and SUDEP included 4 patients who presented with SHE, with four detected *DEPDC5* mutations in these patients. Two patients died of nocturnal SUDEP; however, their cardiac examinations revealed no structural or functional damage (74). This suggests that SUDEP caused by DEPDC5 haploinsufficiency following a genetic mutation may be a consequence of brainstem failure, rather than susceptibility to arrhythmias (74).

Next-generation sequencing technology was used to analyze the genes of 404 cases of focal epilepsy, with two pathogenic mutations of the *NPRL2* gene detected in two patients with familial SHE (p. Leu105Pro and p. Arg34*) and one pathogenic mutation of the *NPRL3* gene (p.Ser279Phefs*52) identified in a familial case of SHE. These mutations were located in protein-coding regions and manifested as various types of focal epilepsy, even within affected families. This was the first report of *NPRL2* and *NPRL3* mutations in SHE, and both exhibited incomplete penetrance (20). Subsequent studies reported the *NPRL2*-related potential pathogenic mutations p.Arg34* and p.Leu105Pro (17, 21). A Chinese case report of a child with sporadic SHE detected a copy number variant and exon 2–11 deletion in *NPRL2*, predicted to be a loss-of-function mutation (22). Currently, 11 *NPRL3* mutations have been detected in patients: p.Ser279Phefs*52, p.Glu508Argfs*46, p.GIn188*, p.Arg424*, p.Try519*, p.Thr51Glyfs*5, p.Y26X, p.Gin399*, R92X, p.Ile255Serfs*28, and exon 5–6 deletion (17, 23–25).

At least 56 GATOR1 mutations related to SHE have been identified to date (17, 20, 21, 23–25, 69, 72–74). Unlike mutations in ligand- or ion-gated channel-associated genes, nonsense mutations are the most common type of GATOR1 mutations (64), which cause the premature appearance of the stop codon, resulting in haploinsufficiency of the GATOR1 protein and even loss of function (20, 71, 75, 76).

Epileptic seizures caused by GATOR1 mutations are related to excessive mTOR signal transduction pathway activation, somatic “second hit” events, and neurodevelopmental GABAergic network defects. Excessive activation of the mTOR signal transduction pathway, abnormal enlargement and malformation of neurons, and excessive dendrite volumes result in abnormal synaptic connections and neuronal ectopy, which constitute the anatomical structural basis of epilepsy (77–79). Moreover, increased excitatory synapse numbers and upregulation of postsynaptic excitatory receptors result in an imbalance between excitatory and inhibitory neuronal activity at the level of ion channels and synapses, generating a functional basis for epileptogenesis (80). Somatic “second hit”: GATOR1 gene mutations carried by neural progenitor cells from germ cells during brain development, causing environmental factor-induced inactivation of the other GATOR1 biallelic gene, which results in somatic cell mosaicism in the brain, loss of GATOR1 function, and epileptic seizures (77). Disease phenotype and severity in patients are related to the timing and location of somatic cell mosaicism; therefore, this may explain the presence or absence of cortical developmental malformations and incomplete genetic penetrance (81, 82). The fine branches of the GABAergic inhibitory network in GATOR1 knockout zebrafish were severely reduced during neural development, independent of mTOR signaling pathway alterations. Therefore, researchers proposed that GABAergic network neurodevelopmental defects may be involved in the development of GATOR1 gene-related epilepsy (83).

### 3.2 TSC complex-related genes

mTOR regulates ion channel expression, synapse formation, and synaptic function in neurons (84). The mTORC1 pathway is crucial for the control of neurotransmission, ion channel expression, and synaptic plasticity in the CNS (85). The tuberous sclerosis complex (TSC), consisting of TSC1, TSC2, and TBC1D7, acts as an upstream repressor of the mTORC1 pathway by inhibiting Rheb, thereby indirectly inhibiting mTORC1 activity in the brain (84, 85). Different experimental studies have demonstrated that pathogenic mutations in TSC1 or TSC2 can lead to overactivation of mTORC1, resulting in altered neuronal intrinsic excitability (hyperexcitation/hypoexcitation) and/or synaptic transmission disorders, which may be the basis for epilepsy onset (84).

The TSC2 pathogenic mutation Arg905GIn was detected in a patient with sporadic SHE who presented with refractory epilepsy. No evidence of TSC-related white matter lesions was present on brain MRI, resulting in a diagnosis of non-focal SHE (19). This mutation is a hotspot mutation in tuberous sclerosis complex. The associated epilepsy phenotype is primary focal epilepsy, and patients with this mutation rarely experience sleep-related events (19). Another case of mild tuberous sclerosis complicated by SHE revealed multiple T2 hyperintensities in the subcortical white matter of the left frontotemporal and parietal regions on brain MRI, with normal cognitive function. Carbamazepine treatment effectively controlled the epileptic seizures in this case. Researchers detected the *TSC1* gene mutation Ser282GInfs*36; however, despite negative paternal gene mutation test results, they were unable to determine whether the mutation was hereditary since the patient’s mother was deceased. Notably, the patient’s maternal aunt had a history of generalized epilepsy (26). The Ser282GInfs*36 mutation detected in TSC1 and the Arg905GIn mutation detected in TSC2 are both pathogenic variants (19, 26). Patients with the Ser282GInfs*36 mutation exhibited structural damage in the cerebral cortex but had a good response to medication, whereas patients with the Arg905GIn mutation did not show structural damage in the cerebral cortex but presented with refractory epilepsy. This suggests that while structural damage may be a risk factor for drug-resistant epilepsy in SHE, additional factors likely contribute to the development and severity of the condition.

Both GATOR1 and TSC complexes are upstream inhibitors of the mTORC1 pathway. Mutations in the three subunit genes of the GATOR1 complex can lead to SHE, with a higher frequency of DEPDC5 gene mutations. Among the genes related to the TSC complex, only one pathogenic mutation in either *TSC1* or *TSC2* was detected in patients with SHE, and no significant difference was observed in the clinical manifestations of epilepsy between patients with SHE with *TSC1* or *TSC2* mutations. SHE with TSC-related gene mutations and cognitive difficulties may be associated with gene mutations and subsequent overactivation of mTORC1, which leads to changes in cytomegalic (oversized) neuronal cell morphology, synaptogenesis changes, and excitatory-inhibitory imbalance (86). In addition, mTORC1 overactivation leads to abnormal cortical lamination and dendritic arborization (branching). Furthermore, repeated seizures and delayed treatment may affect long-term potentiation, short-term plasticity, and connectivity, which may result in neurodevelopmental delay and epilepsy drug resistance (86).

Overall, GATOR1 integrity is crucial for CNS function as any subunit mutation can lead to SHE onset, and the clinical manifestations of SHE caused by them are almost similar. Among all the genes related to SHE, the mutation frequency of GATOR1-related genes was the highest. Therefore, research on GATOR1 gene mutations can contribute insights into precision therapy. Patients with mutations in GATOR1-related genes have higher drug resistance rates, diurnal seizure incidence, and comorbidities including SUDEP. Furthermore, patients often have malformations of cortical development (mainly FCD II), with incomplete penetrance. Developmental delay, cognitive impairment, and intellectual disabilities may also be present in younger patients; however, the symptoms and incidence were milder and lower, respectively, than those of KCNT1 mutant carriers. However, genotype–phenotype correlations cannot be assessed using a single case report of TSC1 and TSC2 gene mutations in SHE. Replication of these phenotypes in larger cohorts and additional functional and *in vivo* behavioral studies are required.

Patients with SHE with mutations in GATOR1 and TSC complex-related genes have a high incidence of FCD, which contributes to the high drug resistance rate; therefore, high-resolution MRI examinations should be repeatedly and carefully reviewed in such patients to detect subtle structural abnormalities, and genetic testing should be performed. Surgical treatment under stereotactic electroencephalogram (EEG) monitoring may benefit patients, particularly those who are drug-resistant. Rapamycin can inhibit the mTOR pathway, and animal experiments have shown that it can significantly prolong the survival time and reduce the enlarged brain tissue and neuronal cell bodies of DEPDC5 knockout mice (87). Early rapamycin treatment may control childhood epileptic seizures and effectively improve developmental delay; nevertheless, further preclinical and clinical studies are required to confirm these effects.

## 4 Other pathogenic genes related to SHE

In addition to the abovementioned genes, mutations in CRH, CaBP4, STX1B, and PRIMA1 can cause SHE. The *CRH* gene encodes corticotropin-releasing hormone (CRH), which is widely distributed in the CNS. CRH is a peptide neurotransmitter and neuromodulator and plays a role in neural circuits outside the hypothalamus, integrating multi-system responses to stress and controlling various behaviors, such as motor activity, anxiety, food intake, sexual behavior, sleep–wake cycles, and learning (27). Researchers have detected two nucleotide mutations (g.1470C > A, g.1166G > C) in the promoter region of the CRH gene in seven patients. These mutations co-segregated with the disease, and *in vitro* experiments showed that these mutations altered protein expression levels (the former caused an increase, whereas the latter caused a decrease) (28). Subsequently, the same research team discovered a heterozygous missense mutation (hpreproCRH p.Pro30Arg) located in the pre-CRH sequence region encoded by the second exon of the CRH gene in an Italian family with SHE. *In vitro* experiments have shown that this mutation causes a short-term decrease in CRH secretion levels, with changes in CRH levels altering sleep sigma activity, increasing epileptic seizure susceptibility, and causing sleep cycle abnormalities (27). This may explain sleep fragmentation and excessive daytime sleepiness in patients with SHE and *CRH* mutations.

The CaBP4 gene encodes Calcium-binding protein 4 (CaBP4), which is a component of the voltage-gated calcium channel Cav1.4 complex in the retina. This gene is mainly expressed in the photoreceptor cells of the retina and controls the release of retinal neurotransmitters (88). Only one *CaBP4* gene mutation associated with SHE has been identified, which was a missense mutation [c.464G > A (p.G155D)] (29) located near the CaBP4 N-terminal EF-hand1Ca^2+^-binding motif. In a four-generation family from southern China, 7 out of 11 family members affected by ADSHE had this mutation. Notably, these individuals did not experience night blindness or visual impairment issues, and neurological examinations and cranial MRI revealed no abnormalities. The average and median ages of onset for these patients were 11 years, and their epilepsy symptoms were effectively relieved by topiramate and levetiracetam treatment. Researchers have speculated that this mutation reduces the activation of ion channels, leading to decreased Ca^2+^ influx, thereby interfering with the release of inhibitory neurotransmitters (29). The research group further confirmed that the p.G155D mutation of the CaBP4 gene reduces the expression of CaBP4 by reducing the stability of the CaBP4 protein, thereby affecting its regulation of neuronal L-type Ca2+ channels and causing changes in neuronal cell excitability (88).

The STX1B gene encodes syntaxin-1B (STX1B), which is a core component of the soluble N-ethylmaleimide-sensitive factor attachment protein receptor (SNARE) complex on the presynaptic membrane of neurons, mediating the exocytosis of synaptic vesicles (89). Studies have shown that pathogenic variants of STX1B result in loss of neurotransmitter release, inhibition of presynaptic vesicle priming, and disruption of vesicle docking at glutamatergic and GABAergic synapses (90). Only one case of SHE with a mutation in *STX1B*, c.106-2A > G, has been reported to date (30). The patient presented with SHE symptoms, mainly pedaling movements of the lower limbs lasting for approximately 30 s, with intact consciousness. The mean arterial pressure increased from 80 to 100 mmHg within 4 s of seizure onset. After the seizure ended, the mean arterial pressure dropped sharply to 54 mmHg for 34 s and subsequently returned to the pre-seizure level. These blood pressure changes were continuously recorded during the seizure, which was not typically performed in other gene mutation-related SHE cases, indicating potential autonomic nervous system instability. The c.106-2A > G mutation may disrupt natural splicing receptor sites, resulting in splicing abnormalities and abnormal protein- or nonsense-mediated mRNA decay; however, the researchers did not verify whether the mutation affected protein function (30). The patient had a paternal history of epilepsy; however, genetic testing of family members was incomplete. Therefore, whether c.106-2A > G occurred as a *de novo* or inherited mutation and whether the patient’s epilepsy was familial or sporadic remains unclear.

The PRIMA1 gene encodes PRIMA1, a type I transmembrane protein rich in proline-rich attachment domain (PRAD). PRIMA1 converts acetylcholinesterase (AChE) into its functional tetrameric form in the synaptic cleft of neuromuscular junctions in the brain and anchors it to neuronal membrane rafts for acetylcholine hydrolysis (91). *PRIMA1* was the first identified autosomal recessive SHE gene. Only one pathogenic mutation has been reported, the homozygous splicing site mutation c.93 + 2 T > C, which causes PRIMA1 to splice immature mRNA, skipping the second exon (the first coding exon). The mutated splice site cannot be recognized, resulting in *PRIMA1* deletion and reduced AChE at the synapse, which leads to acetylcholine accumulation and an enhanced cholinergic response (31). Studies have shown that the c.93 + 2 T > C mutation can result in *PRIMA1* knockout, whereas gained AChR subunit function can lead to enhanced cholinergic responses that may cause severe SHE and intellectual disabilities in affected individuals (31).

Mutations in *PRIMA1* exhibit similar clinical features to those in nAChR mutation-association SHE; however, patients with PRIMA1 mutations often present with urinary incontinence and occasionally with secondary generalized seizures. This indicates that the clinical phenotype of patients becomes more severe after enhancement of the cholinergic response by knocking out *PRIMA1* with a recessive homozygous mutation.

CRH, CaBP4, STX1B, and PRIMA1 mutations have been detected in SHE patients; nevertheless, concluding that these genes are the pathogenic genes of SHE based on individual or a few cases is premature. Larger cohort studies are required to replicate these phenotypes, and additional functional studies and animal experiments are required to understand the exact relationship between these genes and SHE to further clarify the specific pathogenesis and identify therapeutic targets.

## 5 Conclusion

Almost all mutations identified in SHE occurred in protein-encoding genes. These genes contribute to SHE pathogenesis by impacting various biological functions, including ion transport, signal transduction, synaptic transmission, and membrane transport regulation.

Mutations in nAChR-related subunits affect cholinergic projections involved in the sleep–wake cycle, leading to ascending cholinergic pathway overactivation, excessive neuronal synchronization, sleep disruption, and seizure initiation. *KCNT1* mutations result in the gain-of-function of Slo2.2, which is mainly expressed in inhibitory interneurons in the CNS, increasing the excitability of excitatory neurons. *KCNT1* gene mutations have high penetrance, and carriers often exhibit severe symptoms, early disease onset, and severe cognitive issues. *GABRG2* mutations result in reduced synaptic expression and receptor clustering, resulting in channel dysfunction and impairment of the inhibitory GABAergic pathways in the cortical circuitry. However, this gene is more commonly associated with febrile seizures and developmental and epileptic encephalopathy. GATOR1 complex-related gene mutations often lead to hereditary structural lesions, and surgery may be a better treatment option for patients. Gene mutations alter cell signal transduction functions and are more likely to affect developing brains; therefore, epileptic seizures in patients with SHE carrying these gene mutations show certain age correlations, have high drug resistance rates, and are more likely to be combined with SUDEP and diurnal epileptic seizures. Mutations related to *TSC1, TSC2, CRH, CaBP4, STX1B*, and *PRIMA1* are less frequently reported and require replication in larger patient cohorts.

Except for CHRNA4, CHRNB2, and CHRNA2, almost all other genes showed phenotypic heterogeneity, particularly KCNT1 and GATOR1 complex-related genes, which demonstrated a wide pathogenicity spectrum, complicating the study of their specific pathogenic mechanisms in SHE and causing difficulty in clarifying the relationship between genotype and phenotype. Multiple distinct genetic mutations have been identified to individually cause SHE. All identified gene mutations are autosomal dominant, except for the PRIMA1 mutation, which is autosomal recessive. Therefore, 50% of the offspring of patients carrying autosomal dominant gene mutations may be affected, and early genetic interventions may benefit patients.

In summary, the identification and confirmation of candidate pathogenic genes for SHE with the development of genome-wide association studies, whole exome sequencing, whole genome sequencing, and other technologies, combined with *in vitro* functional studies and genetic animal model data, can contribute insights into the pathogenesis and precision therapy for SHE.

## Author contributions

YY: Writing – original draft. JT: Writing – original draft. JZ: Writing – review & editing, Supervision, Methodology. ZX: Supervision, Writing – review & editing, Visualization, Data curation. ZL: Data curation, Investigation, Writing – review & editing.
